# MiR-324-5p inhibition after intrahippocampal kainic acid-induced *status epilepticus* does not prevent epileptogenesis in mice

**DOI:** 10.3389/fneur.2023.1280606

**Published:** 2023-11-16

**Authors:** Amanda M. McGann, Grace C. Westerkamp, Alisha Chalasani, Cole S. K. Danzer, Emma V. Parkins, Valerine Rajathi, Paul S. Horn, Ernest V. Pedapati, Durgesh Tiwari, Steve C. Danzer, Christina Gross

**Affiliations:** ^1^Medical Scientist Training Program, University of Cincinnati College of Medicine, Cincinnati, OH, United States; ^2^Neuroscience Graduate Program, University of Cincinnati College of Medicine, Cincinnati, OH, United States; ^3^Division of Neurology, Cincinnati Children’s Hospital Medical Center, Cincinnati, OH, United States; ^4^Division of Child and Adolescent Psychiatry, Cincinnati Children’s Hospital Medical Center, Cincinnati, OH, United States; ^5^Department of Pediatrics, University of Cincinnati College of Medicine, Cincinnati, OH, United States; ^6^Department of Psychiatry and Behavioral Neuroscience, University of Cincinnati College of Medicine, Cincinnati, OH, United States; ^7^Division of Anesthesia, Cincinnati Children’s Hospital Medical Center, Cincinnati, OH, United States; ^8^Department of Anesthesia, University of Cincinnati College of Medicine, Cincinnati, OH, United States

**Keywords:** epileptogenesis, microRNA, epilepsy, kainic acid, EEG

## Abstract

**Background:**

Acquired epilepsies are caused by an initial brain insult that is followed by epileptogenesis and finally the development of spontaneous recurrent seizures. The mechanisms underlying epileptogenesis are not fully understood. MicroRNAs regulate mRNA translation and stability and are frequently implicated in epilepsy. For example, antagonism of a specific microRNA, miR-324-5p, before brain insult and in a model of chronic epilepsy decreases seizure susceptibility and frequency, respectively. Here, we tested whether antagonism of miR-324-5p during epileptogenesis inhibits the development of epilepsy.

**Methods:**

We used the intrahippocampal kainic acid (IHpKa) model to initiate epileptogenesis in male wild type C57BL/6 J mice aged 6–8 weeks. Twenty-four hours after IHpKa, we administered a miR-324-5p or scrambled control antagomir intracerebroventricularly and implanted cortical surface electrodes for EEG monitoring. EEG data was collected for 28 days and analyzed for seizure frequency and duration, interictal spike activity, and EEG power. Brains were collected for histological analysis.

**Results:**

Histological analysis of brain tissue showed that IHpKa caused characteristic hippocampal damage in most mice regardless of treatment. Antagomir treatment did not affect latency to, frequency, or duration of spontaneous recurrent seizures or interictal spike activity but did alter the temporal development of frequency band-specific EEG power.

**Conclusion:**

These results suggest that miR-324-5p inhibition during epileptogenesis induced by *status epilepticus* does not convey anti-epileptogenic effects despite having subtle effects on EEG frequency bands. Our results highlight the importance of timing of intervention across epilepsy development and suggest that miR-324-5p may act primarily as a proconvulsant rather than a pro-epileptogenic regulator.

## Introduction

Epilepsy affects over 50 million patients worldwide, and one-third of those patients have symptoms that are refractory to current treatment options (1). The development of acquired epilepsies begins with a precipitating brain insult—*status epilepticus* (*SE*), traumatic brain injury, or infection—that is followed by a latent period and finally the development of spontaneous recurrent seizures (SRSs) (2–4). Current treatment primarily addresses the spontaneous recurrent seizures characteristic of developed, chronic epilepsy (2, 5). Epileptogenesis, the latent period following brain insult during which a healthy brain becomes prone to recurrent seizures, is an important timepoint to consider for treatment. Successful intervention during the latent period could prevent or delay the development of epilepsy.

The development of epilepsy is characterized by complex brain alterations that ultimately lead to abnormal brain function and spontaneous seizures (2, 4). MicroRNAs (miRNAs) have been identified as potentially important regulators of these processes (6–8). MiRNAs are short, non-coding RNA sequences that regulate post-transcriptional gene expression via translational suppression or degradation of target messenger RNAs (mRNAs) (9, 10). A single miRNA can target hundreds of mRNAs, thus regulating the expression of hundreds of proteins and influencing large functional networks (11). Their involvement in large functional networks makes miRNAs attractive candidates for therapeutic strategies to treat or prevent disease.

Preclinical studies have identified several miRNAs that modulate seizure susceptibility and can be targeted to improve seizure-related outcomes. These include but are not limited to miR-146a (12–14), miR-134 (15–19), miR-135a (20), and miR-324-5p (21–24). We found miR-324-5p to be a particularly interesting target for investigation in epileptogenesis because antagonism of miR-324-5p has been shown to reduce seizures at two time points of disease progression: miR-324-5p inhibition before brain insult or in chronic epilepsy reduces seizure susceptibility and improves epilepsy phenotype, respectively (21, 23). To further evaluate the potential therapeutic value of miR-324-5p inhibition, it is important to investigate whether antagomirs to miR-324-5p have anti-epileptogenic and thus disease-modifying effects when administered during epileptogenesis. Notably, miR-324-5p exerts its effects in part by targeting and translationally suppressing the mRNA coding for the A-type potassium channel Kv4.2 (21, 23). The targeting and downregulation of Kv4.2, specifically, suggests that antagonism of miR-324-5p inhibits seizure activity primarily by acting as an anti-convulsant; however, since one miRNA can target hundreds of mRNA (11) and miR-324-5p has several confirmed targets (25–30), it is possible that antagonism of miR-324-5p could serve not only as an anti-convulsant but also an anti-epileptogenic therapy. This is in line with our published findings showing that administration of miR-324-5p antagomirs in Kv4.2 knockout mice abolishes the effect on latency to kainic acid-induced seizure, while overall EEG power after kainic acid is still reduced, suggesting a seizure-dampening effect mediated by other miR-324-5p targets (21). Importantly, many studies have successfully targeted miRNAs before brain insult and in chronic epilepsy, but very few have assessed their role during the latent period, epileptogenesis (31).

To address this gap in knowledge, we investigated the effect of miR-324-5p antagonism during epileptogenesis. Using the intrahippocampal kainic acid model of mesial temporal lobe epilepsy to initiate epileptogenesis in mice, we tested the hypothesis that miR-324-5p inhibition during epileptogenesis delays or prevents the development of epilepsy. Our results showed that miR-324-5p inhibition during epileptogenesis does not alter hippocampal morphology, onset or frequency of spontaneous recurrent seizures, or frequency of interictal spikes in the first 4 weeks following brain insult. Inhibition of miR-324-5p did, however, alter spectral EEG power and how the frequencies of select power bands changed over time. Overall, we conclude that miR-324-5p inhibition shortly after *SE* before the occurrence of spontaneous seizures does not confer anti-epileptogenic effects.

## Materials and methods

### Mice

All experiments were conducted in accordance with the Institutional Animal Care and Use Committee of CCHMC and followed National Institute of Health Guidelines for the Care and Use of Laboratory Animals. All mice were housed in standard cages on a 14/10 light/dark cycle with access to food (Cat# 5010, LabDiet) and water *ad libitum*. All experiments were conducted using male C57BL/6 J WT mice obtained from Jackson Laboratories (Stock #000664, RRID:IMSR_JAX:000664) or their offspring bred in-house. Mice were housed in sex-matched groups of up to four per cage. After surgeries, mice were single housed. Although single housing has been shown to affect disease severity in rodent epilepsy models (32), it was necessary in this study for EEG data collection. A total of 45 mice from 17 different litters were used in the study. Forty-three mice were intrahippocampally injected with kainic acid and 2 mice with saline. All 45 mice received EEG transmitter implants and intracerebroventricular antagomir treatment; 22 mice from 15 different litters received miR-324-5p antagomir and 23 mice from 16 different litters received scrambled control antagomir. Eleven mice were removed from the study due to poor EEG signal (n = 1), clerical issues in treatment recording (n = 2), and premature deaths unrelated to epilepsy model (n = 8). Perfused brain tissue was collected for immunohistochemical analysis for 20 mice. Inclusion criteria for individual analyses are listed under “Statistics.”

### Drugs, antibodies, and antagomirs

Kainic acid (Cat. No. 7065 Tocris Biosciences, Bristol, United Kingdom) was dissolved in 0.9% sodium chloride to achieve a concentration of 4 mg/mL. Mouse monoclonal anti-NeuN antibody (1:250, Cat. #ab177487, RRID:AB_2532109, Abcam, Cambridge, MA, UK) and Alexa Fluor 488 donkey anti-mouse secondary antibody (1:200, Cat #715–545–150, RRID: AB_2340846, Jackson ImmunoResearch Laboratories, West Grove, PA) were used for fluorescent immunohistochemistry. The antagomirs were locked-nucleic acid-modified, custom-made *in vivo* inhibitors from Qiagen (Hilden, Germany). Both miR-324-5p and scrambled antagomirs were 14–15 nucleotides in length with a partial phosphorothioate backbone and no cholesterol tag (due to problems with synthesis and solubility). Antagomirs were specific for miR-324-5p (Cat. #339146 YCI02000086-FDA, sequence 5′–3′: ACCAATGCCCTAGG) or a scrambled control that does not target any miRNAs in mammals (Cat. #339146 YCI0199810-FDA, sequence 5′–3′: ACGTCTATACGCCCA). The functional activity of the miR-324-5p antagomir has been confirmed previously in our lab and those of collaborators at 24 h (21) and 7–14 days post-injection (23). Additionally, antagomirs specific to other miRNAs but with the same molecular characteristics have been shown to remain detectable and functionally active for up to 1 month (15).

### Epilepsy model (intrahippocampal kainic acid)

Six- to 8-week-old mice received a unilateral, right-sided intrahippocampal injection of 64 nL 4 mg/mL kainic acid (18.8 mM, 0.256 μg) dissolved in 0.9% sodium chloride or 64 nL 0.9% sodium chloride (saline; Hospira Inc., Lake Forest, IL) (33–36). All injections occurred between 8:00 a.m. and 1:00 p.m. (ZT2-ZT7). Anesthesia was induced using an inhaled mixture of 3% isoflurane and 1.5% oxygen, then maintained using a mixture of 1.5% isoflurane and 1.5% oxygen. Mice were administered 1 mg/kg carprofen at the beginning of surgical procedure and once daily for 3 days following procedure for analgesia. Throughout surgical procedure (20–30 min), mice were kept on a warmer at 37.2°C (Rodent Warmer X1 Item 53,800 M, Stoelting, IL, United States). One burr hole (less than 1 mm in diameter) was drilled at the following stereotaxic coordinates from bregma: AP = −2.0 mm, ML = +1.2 mm (right-sided). A 5 μL Hamilton syringe designed specifically for rodent brain injections (Ref #65460–02, 5 μL, Neuros Syringe, Model 75 RN, 33-gauge, Point Style 3, Hamilton Company, United States) was lowered from the dura mater 1.4 mm into the right hippocampus (DV = −1.4 mm). Kainic acid/saline was administered slowly over 1 min at a rate of 64 nL/min. The needle was left in place for 5 min to allow diffusion of the injected volume, then withdrawn over 1.5 min. Post injection, the open skin incision was closed with surgical sutures (Covidien, Medtronic, Minneapolis, MN). An antibiotic ointment (Globe Chemical Company, Marietta, GA) was applied over the sutures. Mice were administered 1 mL 0.9% sodium chloride intraperitoneally and monitored for recovery.

### Intracerebroventricular injection of microRNA antagomir

One day following intrahippocampal kainic acid/saline injection and EEG transmitter implantation (if applicable), mice received a unilateral, right-sided intracerebroventricular (ICV) injection of miR-324-5p antagomir or scrambled control [0.5 nmol in 2 μL sterile artificial cerebrospinal fluid (ACSF)] following established protocols (21, 23). All injections occurred between 8:00 a.m. and 1:00 p.m. (ZT2-ZT7). Anesthesia was induced using an inhaled mixture of 3% isoflurane and 1.5% oxygen, then maintained using a mixture of 1.5% isoflurane and 1.5% oxygen. Mice were administered 1 mg/kg carprofen at the beginning of surgical procedure and once daily for 3 days following procedure for analgesia. Throughout surgical procedure (25–35 min), mice were kept on a warmer at 37.2°C (Rodent Warmer X1 Item 53800 M, Stoelting, IL, United States). One burr hole (less than 1 mm in diameter) was drilled at the following stereotaxic coordinates from bregma: AP = −0.3 mm, ML = +1.0 mm. A 5 μL Hamilton syringe designed specifically for rodent brain injections (Ref #65460-02, 5 μL, Neuros Syringe, Model 75 RN, 33 gauge, Point Style 3, Hamilton Company, United States) was lowered from the dura mater 2 mm into the right ventricle (DV = −2.0 mm). A volume of 2 μL antagomir/scrambled control was administered slowly over 2 min at a rate of 1,000 nL/min. The needle was left in place for 10 min to allow diffusion of the injected volume, then withdrawn over 2 min. Post injection, the open skin incision was closed with surgical sutures (Covidien, Medtronic, Minneapolis, MN) and GLUture (Zoetis Inc., Kalamazoo, MI). An antibiotic ointment (RARO, Hawthorne, NY) was applied over the sutures. Mice were administered 1 mL saline intraperitoneally and monitored for recovery.

### EEG transmitter implantation

Mice were implanted with cortical electrodes for EEG monitoring on the day of kainic acid or the day of antagomir injection (1 day after kainic acid injection) during the respective surgeries. Two burr holes (less than 1 mm in diameter) were drilled in the skull (Bregma; AP = −3.0 mm, L = ± 2.0 mm) for placement of EEG electrodes. The transmitter (ETA-F10, 1.6 g weight, 1.1 cc vol, Data Sciences International, St. Paul, MN) was placed subcutaneously behind the neck. Approximately 1 mm length of exposed wire on each electrode was placed into each burr hole. Leads were positioned horizontally below the skull but above the dura mater, and care was taken not to penetrate the dura mater. Post transmitter implantation, the open skin incision was closed with surgical sutures (Covidien, Medtronic, Minneapolis, MN). An antibiotic ointment (Globe Chemical Company, Marietta, GA) was applied over the sutures. Mice were administered 1 mL saline intraperitoneally and monitored for recovery.

### Continuous video-EEG recording

Mice were single-housed in static cages on wireless receiver plates (RPC-1, Data Sciences International [DSI™], St. Paul, MN). DATAQUEST A.R.T. software was used for EEG data received from the telemetry system sampled at 500 Hz, which provided readouts for frequencies between 1 and 200 Hz (maximum sampling rate of the wireless transmitter ETA-F10). Video was continuously recorded (Axis 221, Axis Communication, Lund, Sweden) in parallel and synchronized with EEG signal. Video-EEG data was collected for 28 days (or until mouse death/euthanasia) and analyzed using *NeuroScore*™ Version 3.4.0.21113 (DSI™).

### Confirmation of initial status epilepticus and analysis of spontaneous recurrent seizures

Initial *SE* was confirmed behaviorally using the Racine Scale (37) and electrographically. IHpKa induced stage 5 convulsive seizures that recurred over a period of several hours with few or no periods of normal behavior. This behavior was accompanied by hours-long high amplitude/high frequency EEG activity (38). In 27 of the 32 mice injected with kainic acid and used in analysis, we confirmed *SE* in the 24 h following kainic acid injection by EEG-video (10 mice), behaviorally through analysis of video recordings (16 mice), or by direct observation by the experimenter (1 mouse). The occurrence of initial *SE* in all 27 mice monitored during the first 24 h confirmed the robustness of our model. Consequently, we opted to include an additional 5 mice in subsequent analyses although there were no video or EEG data available within the immediate 24 h following IHpKa to confirm *SE*. Spontaneous recurrent seizures were identified manually by electrographic changes on EEG and confirmed by behavioral changes (Racine Scale) in video analysis if available. A seizure was defined electrographically as an abrupt increase in the frequency and amplitude of the EEG signal (at least 2× baseline) with a minimum duration of 10 s. Seizure termination was indicated by return of EEG signal to or below baseline and cessation of seizure behavior. Due to both technical issues and user error, we could not collect continuous 24/7 video-EEG data for 28 consecutive days in all mice (EEG data availability shown in Supplementary Figure 1). Therefore, mice were only included in analyses of days for which they had complete EEG data (i.e., a mouse with missing EEG 1–5 a.m. on Day 8 is excluded from analyses involving Day 8). Additional inclusion criteria are described in results and figure legends.

### Electrographic spike analysis

EEG spikes were analyzed using a spike detector module (dynamic threshold) in *NeuroScore*™ Version 3.4.0.21113 (DSI™) followed by manual review to exclude artifacts. A spike was defined as having a duration of 1-70 ms, a threshold ratio of 3x baseline and a maximum ratio of 10× baseline, and a minimum amplitude of 100 μV. Spike trains were defined as having a minimum duration of 5 ms with a minimum number of 4 spikes within 80–5,000 ms (39, 40). EEG artifacts due to movement or grooming were confirmed via video recording and removed. We conducted spike analysis on 1-h segments of video-EEG data between 12 p.m. and 3 p.m. (ZT6-ZT9) over 3 days at an early time point (days 7–9) and 3 days at a later time point (days 25–27), and only selected intervals that were at least 1 h before or after spontaneous seizure activity. Spike and spike train frequency were averaged over 3 data points for early and late timepoints (6 total data points).

### Spectral power calculations

Spectral power was calculated during an early time point (days 7–9) and a later time point (days 25–27). Three five-minute segments of video-EEG data were selected for each mouse during both a.m. (dark phase, 12–3 a.m., ZT18-ZT21) and p.m. (light phase, 12–3 p.m., ZT6-ZT9) time periods on each day. Segments were selected in which mice were not moving to minimize EEG background and capture accurate signal. All segments were at least 1 h away from spontaneous seizure activity. Spectral power was calculated using an established protocol (41). Data was exported in raw format from the acquisition amplifier and imported into MATLAB (Version 2021b, The MathWorks Inc., Natick, MA, United States). Raw data were visually inspected to exclude segments with movement/muscle artifact contamination; zero files were excluded. Absolute and relative power were calculated using EEGLAB v2022.0, with standard frequency band definitions: delta (2–4 Hz), theta (4–10 Hz), alpha (10–13 Hz), beta (13–30 Hz), gamma1 (30–55 Hz), and gamma2 (65–100 Hz). The segments (2 s) underwent Fast Fourier transformation (FFT) using a Hanning window with 0.5 Hz bin resolution, yielding absolute power (μV^2^/Hz) data across 1–100 Hz frequency range. To obtain relative power, the cumulative absolute power (μV^2^/Hz) in each band was divided by the total power across all bands and averaged across the available trials.

### Visualization of spectral power

Within each day (early: 7–9; late: 25–27) and time of day (a.m. vs. p.m.), the measurements from three 5-min EEG segments were averaged. This resulted in one a.m. and one p.m. value for each day. Each timepoint (early vs. late) contained 3 days, and the average values for each day were graphed as replicates. Note that the statistical analysis (see below) considered these three values (per early/late timepoint) as replicates from the same mouse. Boxplots displaying the median and interquartile range by frequency band were created in R.

### Statistical modeling of spectral power

A repeated measures linear mixed model was fit for each power measure. Within each day (early: 7–9; late: 25–27) and time of day (a.m. vs. p.m.), measurements from three 5-min EEG segments were averaged. This resulted in 1 a.m. and 1 p.m. value for each day. Each timepoint (early vs. late) contained 3 days, and the average values for each day were treated as replicates. Averaging 5-min segments within each day allowed for robust representation of individual mouse EEG power by day, while treating each day as a replicate accounted for the within-subject variability of this model. The compound symmetric covariance structure was used for the random effect, the individual mouse. The independent variable was the treatment group, and the covariates were timepoint (early vs. late) and time of day (a.m. vs. p.m.). All second order interaction terms were included with the three variables as well as the third order interaction term. The distribution used for each power measure was either Normal or Log-Normal, depending on the residual profiles. Transformations are indicated in the Results section. All analyses were conducted using SAS ® version 9.4 (SAS Institute Inc., Cary, NC).

### Immunohistochemistry

Twenty-eight days after IHpKa, mice were deeply anesthetized using 320 mg/kg pentobarbital, then intracardially perfused with 4% paraformaldehyde (PFA). Whole brains were removed and preserved in 4% PFA overnight at 4°C, then cryoprotected in a sucrose solution (24 h in 10% sucrose, 72 h in 30% sucrose at 4°C) and cryopreserved at −80°C. Brains were embedded in Tissue-Tek OCT compound and serially sectioned at 25 μm at Bregma = −1.06–−3.28 mm. For immunohistochemistry, slide-mounted tissue was thawed for 10 min, then washed in 1X tris-buffered saline (TBS). It was treated with 0.8% sodium borohydride solution for 10 min, followed by washes in 1X TBS. We performed antigen retrieval by incubating slides in 0.01 M sodium citrate, pH 6.0 at 100°C for 90s, then allowed slides to cool. Following additional washes in 1X TBS, tissue was permeabilized in 0.5% Triton X-100/TBS for 20 min. Following additional washes in 1X TBS, slides were incubated in a non-specific blocking buffer (10% donkey serum, 1% Triton X, 1X TBS) for 1 h at room temperature. Slides were then incubated in primary antibody solution (2% donkey serum, 1% Triton X, 1X TBS) with a mouse monoclonal anti-NeuN antibody (1:250) overnight at room temperature. The following day, slides were washed with 1X TBS then incubated in secondary antibody solution (2% donkey serum, 1% Triton X, 1X TBS) with Alexa Fluor 488 donkey anti-mouse secondary antibody (1:200) for 1 h at room temperature. Following additional washes in 1X TBS, slides were mounted using ProLong Diamond Antifade Mountant (Invitrogen, Waltham, MA) and stored at −80°C. Tissue was imaged on a Nikon A1R inverted confocal microscope using Nikon Elements software (Tokyo, Japan, RRID: SCR_014329). Tiled images of bilateral hippocampi were collected at 4X magnification with 25μm z-stacks (2.5μm steps). Z-stacks were compressed into maximum image projections for analysis. Hippocampal measurements were obtained using ImageJ software (RRID: SCR_003070). Up to four serial sections (average = 3 sections, range = 1–4 sections) per mouse were visually assessed for the presence of morphological changes across Bregma levels −1.06 mm to −3.28 mm. A single section per mouse between Bregma levels −1.22 mm and − 2.18 mm (IHpKa injection at Bregma = −2.0 mm) was used for quantification of morphological changes. Representative images of ImageJ tracings used to obtain measurements are shown in the respective figure. All tracings/measurements were completed by the same experimenter who was blind to the condition, then reviewed for accuracy by a second experimenter.

### Statistical analyses and inclusion/exclusion criteria

**Figure 1:**
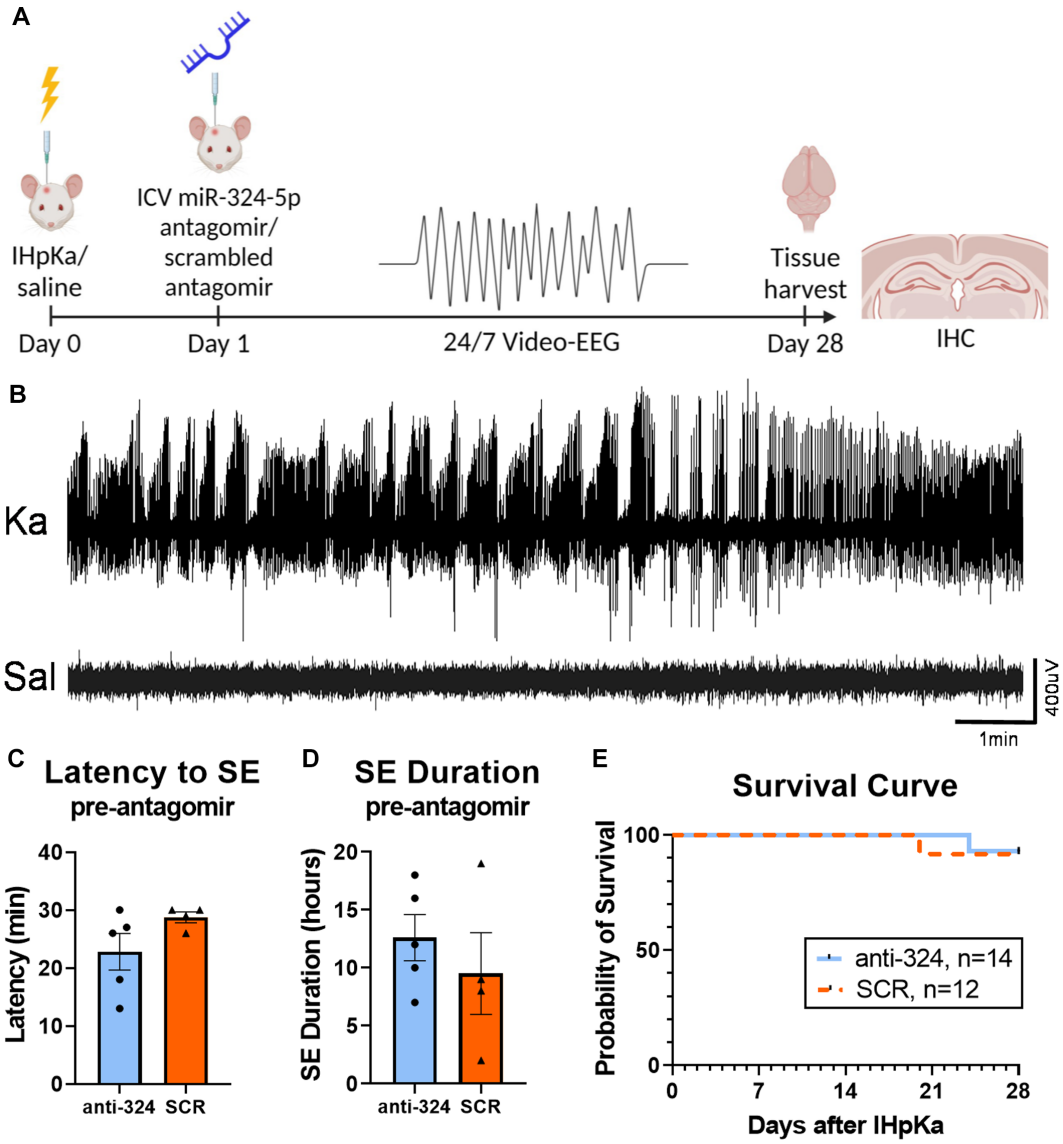
Intrahippocampal kainic acid injection reliably induces *status epilepticus* and initiates epileptogenesis. **(A)** Timeline. On day 0, mice received intrahippocampal kainic acid (IHpKa) or saline injections. Twenty-four hours later, on day 1, mice were intracerebroventricularly injected with an antagomir to miR-324-5p (anti-324) or a scrambled control (SCR). All mice were injected into the right hippocampus and ventricle, respectively. Mice were implanted with EEG transmitters on either day 0 or day 1. Twenty-four/seven video-EEG data was collected for 28 days, at which time brain tissue was collected for immunohistochemical analysis. **(B)** Representative EEG tracings from mice approximately 7 h after IHpKa or saline injection. Mice injected with kainic acid reliably exhibited behavioral and electrographic *status epilepticus* (*SE*), while saline-injected mice did not show epileptic activity. **(C,D)** EEG analysis during the 24 h following IHpKa (prior to antagomir treatment) in a subset of mice shows that initial *SE* severity was similar between treatment groups with no difference in the latency to initial *SE* [**C**, unpaired *t*-test: *t* = 1.62, *p* = 0.15, *n*(anti-324) = 5, *n*(SCR) = 4] or duration of initial *SE* [**D**, unpaired *t*-test: *t* = 0.81, *p* = 0.44, *n*(anti-324) = 5, *n*(SCR) = 4]. Bar graphs represent mean ± SEM. Points represent individual mice. **(E)** Antagomir treatment group had no effect on survival [Mantel-Cox test: X^2^ = 0.25, *p* = 0.62, *n*(anti-324) = 14, *n*(SCR) = 12]. Mice that died from poor health not related to epilepsy phenotype (e.g., post-surgical weight loss, skin lesions) and mice that were sacrificed for other reasons (e.g., loosening of electrodes) were not included in the survival analysis.

The goal of this study was to assess the effect of miR-324-5p antagonism during epileptogenesis in a mouse model of epilepsy. Statistical methods were selected for experimental design with random sampling. Apart from preliminary group assignment assessments and assessment of seizure duration at specific timepoints (not all mice had seizures to include in analysis), we used at least 6 mice per condition from 5 or more different litters for each experimental group as indicated under “Mice.” Experimental groups were randomly assigned, and investigators were blinded throughout experiment and analyses. Apart from spectral power analysis, data were analyzed and visualized using GraphPad Prism v9.2.0. Significance level was set to α = 0.05, and *p* < 0.05 was considered significant. Data sets were tested for normality using the Shapiro–Wilk test, and the appropriate parametric or non-parametric tests were used. Statistical outliers were identified and removed using the Rout method, and number of statistical outliers (if any) are indicated in the figure legends. Statistical tests used and their results as well as sample sizes are indicated in the results and/or figure legends. We were not able to collect 28 days of continuous video/EEG data for all 34 mice in this study for various reasons including equipment failure, technical errors, early death, or early termination (e.g., due to loosened electrode wires). Therefore, not all mice could be used in all statistical analyses. A detailed list of days with available EEG data for each mouse in this study is shown in Supplementary Figure 1. The following stringent inclusion and exclusion criteria for each analysis were implemented (also listed in figure legends):

Figure 1: Figures 1C,D include mice that received EEG transmitter implants on day 0 for analysis of the first 24 h after IHpKa [*n*(324) = 5, *n*(SCR) = 4]. Figure 1E includes mice that received EEG transmitter implants on days 0 or 1. Figure 1E does not include mice that were sacrificed for reasons not related to epilepsy model [e.g., loosening of electrodes; *n*(total) = 6].

**Figure 2:**
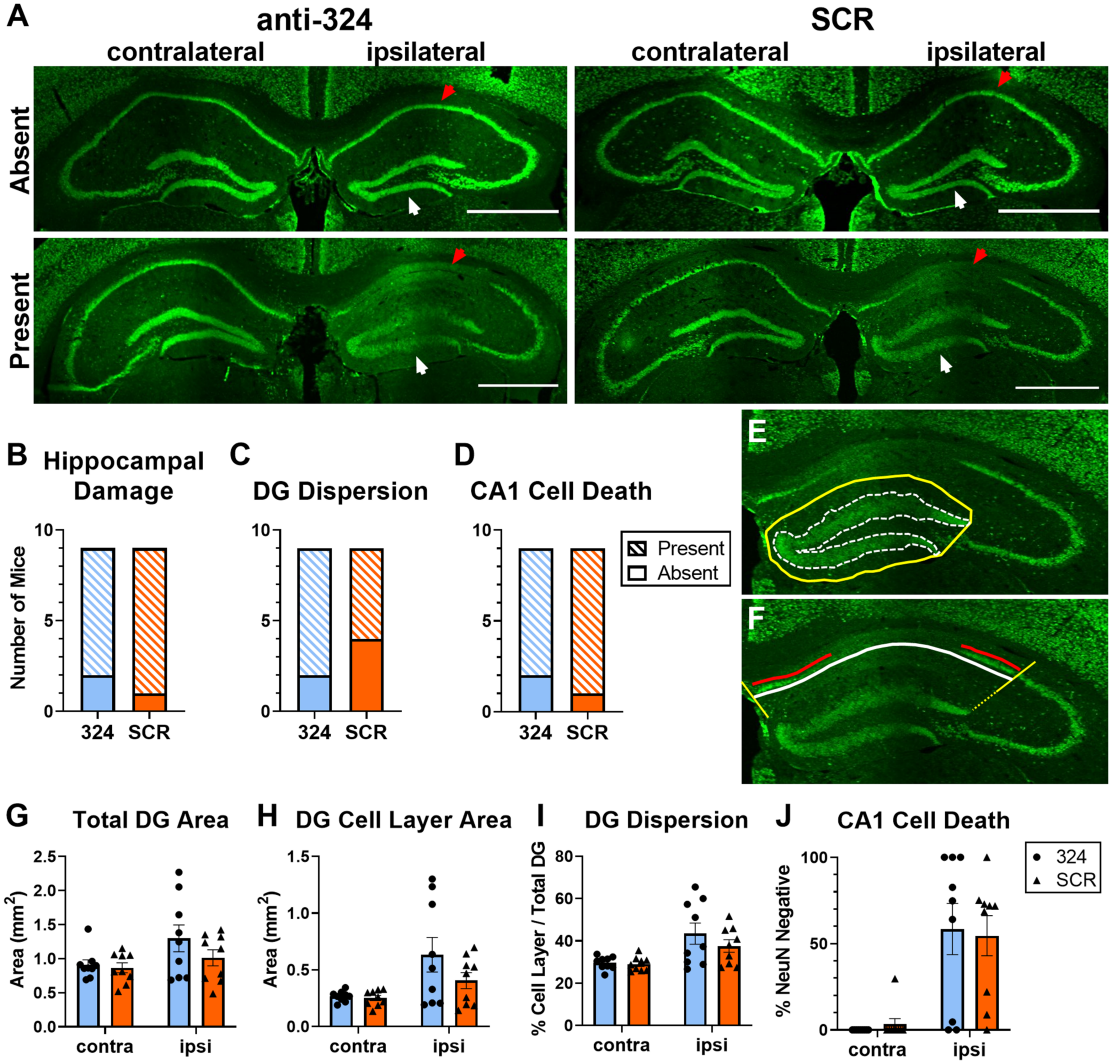
Antagomir treatment does not prevent granule cell dispersion or pyramidal cell loss. **(A)** Representative images of bilateral mouse hippocampal tissue collected on day 28 and immunohistochemically labeled with anti-NeuN antibody. Up to four serial sections (average = 3 sections, range = 1–4 sections) per mouse between Bregma levels −1.06 mm and − 3.28 mm were visually assessed for the presence of morphological changes **(B–D)**. A single brain section between Bregma levels −1.22 mm and − 2.18 mm (IHpKa injection Bregma = −2.0 mm) was quantified for each mouse **(G–J)**. Ipsilateral and contralateral refer to side of kainic acid injection. “Absent” indicates the absence of morphological changes and “Present” indicates the presence of morphological changes. Some mice in each antagomir treatment group exhibited no morphological changes (top), while the majority exhibited dentate granule (DG) cell dispersion and CA1 cell death (bottom). White arrows indicate DG cell layer; red arrows indicate CA1 cell layer. Scale = 1 mm. **(B–D)** Antagomir treatment had no effect on the proportion of mice that exhibited any hippocampal damage [DG dispersion and/or CA1 cell death; **(B)**, Fisher’s exact test: *p* > 0.99, *n*(anti-324) = 9, *n*(SCR) = 9], DG dispersion [**C**, Fisher’s exact test: *p* = 0.62, *n*(anti-324) = 9, *n*(SCR) = 9], or CA1 cell death [**D**, Fisher’s exact test: *p* > 0.99, *n*(anti-324) = 9, *n*(SCR) = 9]. **(E,F)** Representative tracings to illustrate how hippocampal damage was quantified. The areas of the total dentate gyrus (yellow, solid line) and DG cell layer (white, dotted line) were used to estimate DG dispersion **(E)**. To estimate CA1 cell death, the total CA1 cell layer (white line) and NeuN-positive CA1 cell layer (red line, living cells) were quantified **(F)**. Boundaries on the CA1 cell layer were determined by drawing lines at a 135° angle from the medial aspect of the DG cell layer and a 45° angle from the lateral aspect of the dorsal DG cell layer (yellow lines). Figure tracings positioned for better visibility. **(G–I)** Independent of antagomir treatment, the total area of the dentate gyrus ipsilateral to kainic acid injection was larger than that of the contralateral dentate gyrus [**G**, two-way ANOVA: *p*(interaction) = 0.26, *p*(side) = 0.02, *p*(treatment) = 0.27, *n* = 9 mice/group], the area of the ipsilateral DG cell layer was larger than that of the contralateral [**H**, two-way ANOVA: *p*(interaction) = 0.19, *p*(side) = 0.004, *p*(treatment) = 0.20, *n* = 9 mice/group], and DG cell dispersion was greater in the ipsilateral than contralateral DG [**I**, two-way ANOVA: *p*(interaction) = 0.34, *p*(side) = 0.0007, *p*(treatment) = 0.35, *n* = 9 mice/group]. **(J)** CA1 cell death was greater in the hippocampus ipsilateral to kainic acid injection than in the contralateral, independent of antagomir treatment [two-way ANOVA: *p*(interaction) = 0.71, *p*(side) < 0.0001, *p*(treatment) = 0.98, *n* = 9 mice/group]. Supplementary Figure 2 shows the effect of antagomir treatment across the dorsoventral brain axis.

Figure 2: Figure 2 only includes mice for which perfused brain tissue was collected on day 28 [*n*(324) = 9, *n*(SCR) = 9].

**Figure 3:**
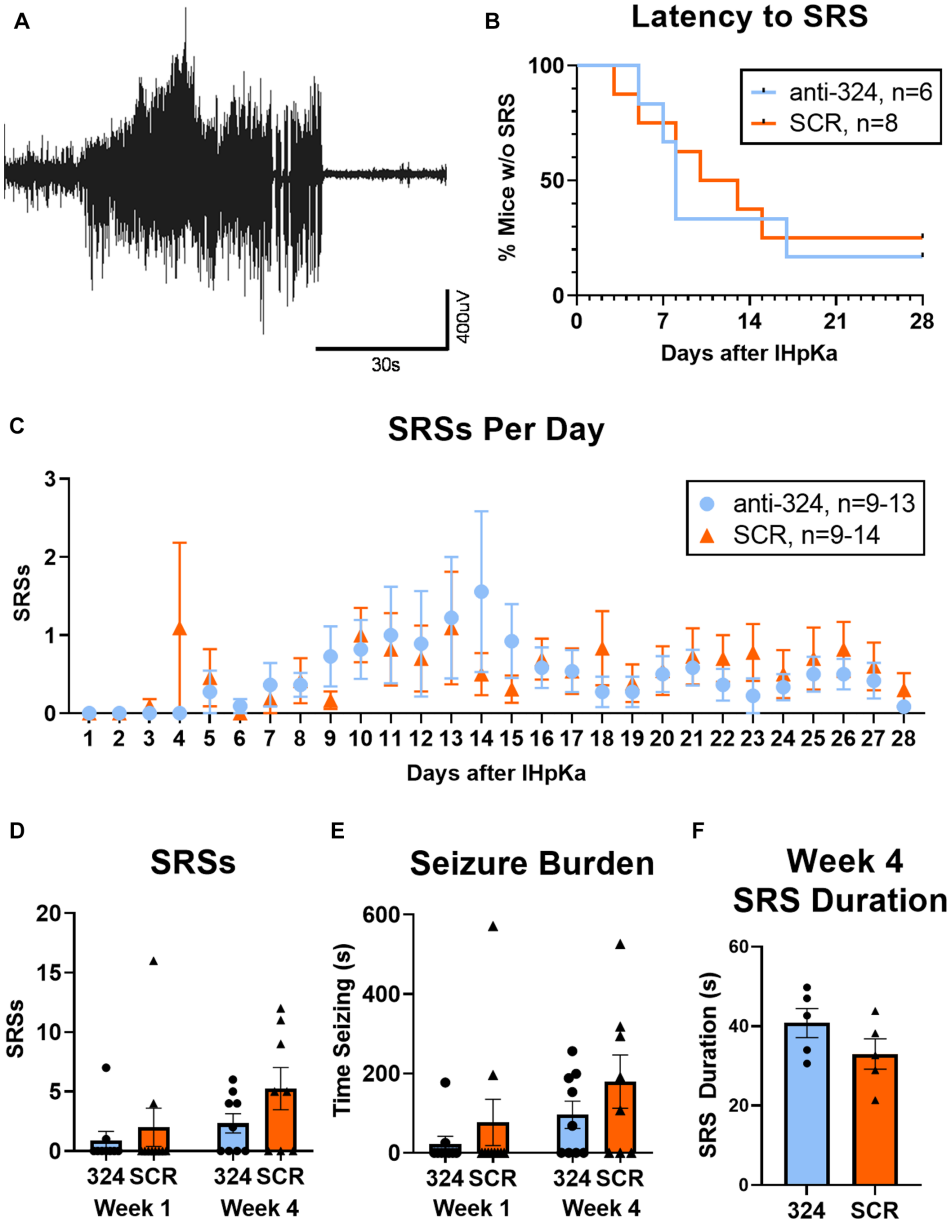
MiR-324-5p inhibition during epileptogenesis does not alter seizure incidence. **(A)** Representative electroencephalographic SRS. **(B)** Antagomir treatment had no effect on seizure latency [Mantel-Cox test: X^2^ = 0.10, *p* = 0.76, *n*(anti-324) = 6, *n*(SCR) = 8]. Analysis of seizure latency only included mice that had complete EEG data from antagomir treatment on day 1 to first SRS. **(C)** Antagomir treatment had no effect on daily SRS frequency within 28 days of IHpKa [Two-way ANOVA: *p*(interaction) = 0.96, *p*(day) = 0.15, *p*(treatment) = 0.57, *n*(anti-324) = 9–13, *n*(SCR) = 9–14]. One outlier was identified and excluded on day 14 (mouse had 21 SRSs) and one on day 15 (mouse had 49 SRSs). Outliers were two different mice; both had been treated with SCR. Analysis of daily SRS frequency only included SRS data for mice with 24 h of uninterrupted EEG recording on individual days. Points represent group means ± SEM. **(D)** Antagomir treatment had no effect on total SRSs in week 1 and week 4 [two-way ANOVA: *p*(interaction) = 0.50, *p*(week) = 0.08, *p*(treatment) = 0.14, *n*(anti-324, week 1) = 10, *n*(anti-324, week 4) = 10, *n*(SCR, week 1) = 10, *n*(SCR, week 4) = 8]. Weekly SRS analysis only included mice with complete 24/7 EEG data for the duration of each week. **(E)** Antagomir treatment had no effect on seizure burden in week 1 and week 4 [two-way ANOVA: *p*(interaction) = 0.77, *p*(week) = 0.08, *p*(treatment) = 0.17, *n*(anti-324, week 1) = 10, *n*(anti-324, week 4) = 10, *n*(SCR, week 1) = 10, *n*(SCR, week 4) = 8]. **(F)** Antagomir treatment had no effect on SRS duration in week 4 [unpaired *t*-test: *t* = 1.46 *p* = 0.18, *n*(anti-324) = 5, *n*(SCR) = 5]. Analysis of SRS duration only included mice that had SRSs in week 4. Bar graphs represent group mean ± SEM. Points represent individual mice.

Figure 3: Figure 3B only includes mice that had continuous EEG data from antagomir treatment on day 1 to first SRS [*n*(324) = 6, *n*(SCR) = 8]. Figure 3C only includes mice with 24 h of continuous EEG recording on each individual day [i.e., if a mouse was missing 2 h of EEG data on day 8, it was not included in analysis of seizure frequency on day 8; *n*(324) = 9–13, *n*(SCR) = 9–14]. Figures 3D,E only include mice with continuous 24/7 EEG data for the duration of each week 1 and/or week 4 [*n*(324, week 1) = 10, *n*(324, week 4) = 10, *n*(SCR, week 1) = 10, *n*(SCR, week 4) = 8]. Figure 3F includes only mice that had SRSs during week 4 [*n*(324) = 5, *n*(SCR) = 5].

**Figure 4:**
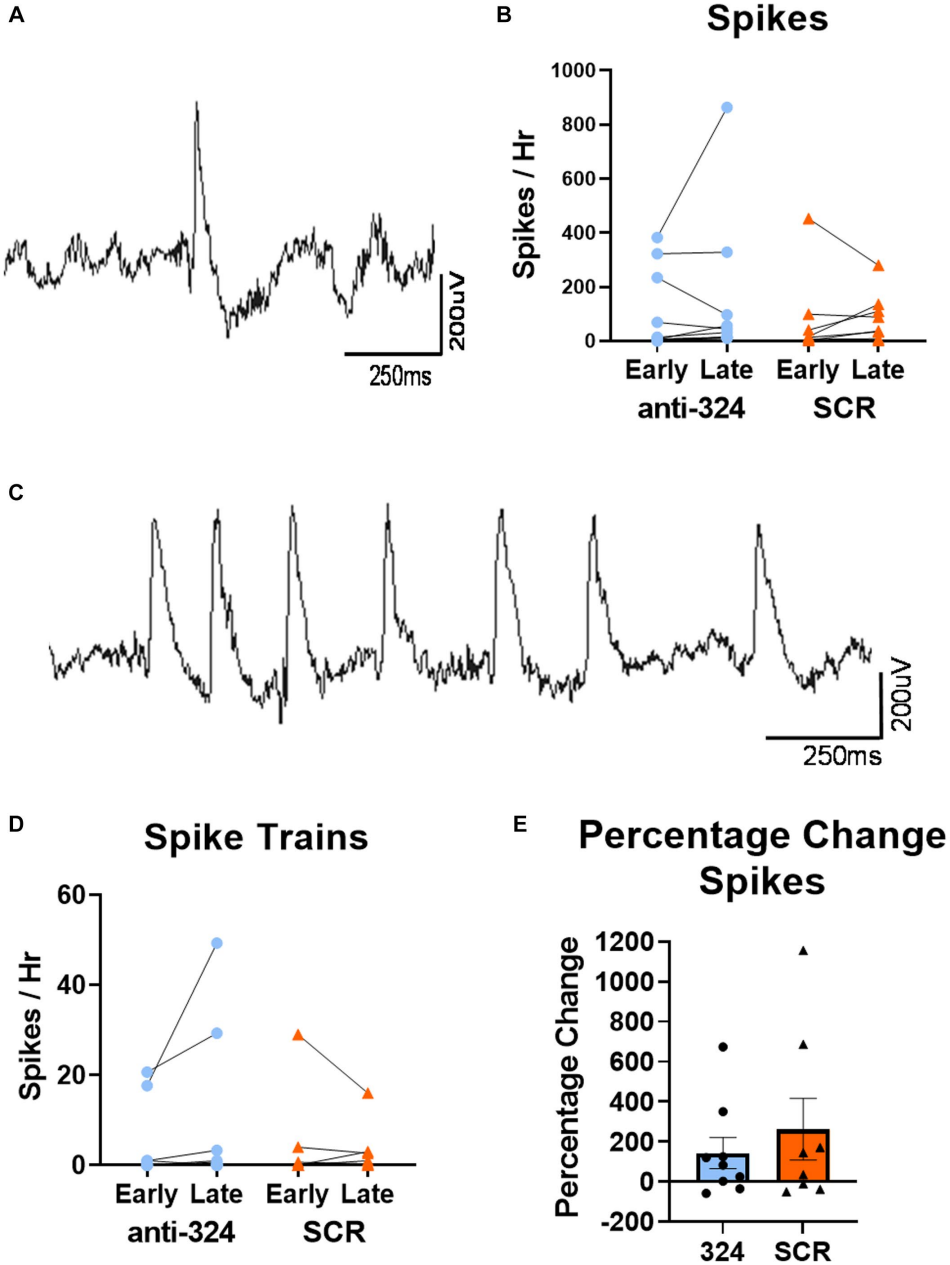
MiR-324-5p inhibition during epileptogenesis does not alter frequency of interictal spikes or spike trains. **(A)** Representative image of single epileptiform spike. **(C)** Representative image of spike train. **(B,D)** Early timepoint refers to days 7–9 and late timepoint refers to days 25–27 after IHpKa. Analysis only included mice with continuous EEG data during both timepoints. Spikes and spike trains were quantified for 1 h on each of 3 days within each timepoint. Points on bar graphs represent the mean value of spikes or spike trains over 3 days in each individual mouse. Bars represent group mean ± SEM. Antagomir treatment had no effect on frequency of spikes [**B**, two-way RM ANOVA: *p*(interaction) = 0.59, *p*(timepoint) = 0.44, *p*(treatment) = 0.61, *n*(anti-324) = 10, *n*(SCR) = 8] or spike trains [**D**, two-way RM ANOVA: *p*(interaction) = 0.17, *p*(timepoint) = 0.45, *p*(treatment) = 0.60, *n*(anti-324) = 10, *n*(SCR) = 8]. **(E)** Antagomir treatment had no effect on within-mouse percentage change in spikes between early and late timepoints [Mann–Whitney test: *p* = 0.74, *n*(anti-324) = 10, *n*(SCR) = 8]. Bar graphs represent group mean ± SEM. Points represent individual mice.

**Figure 5:**
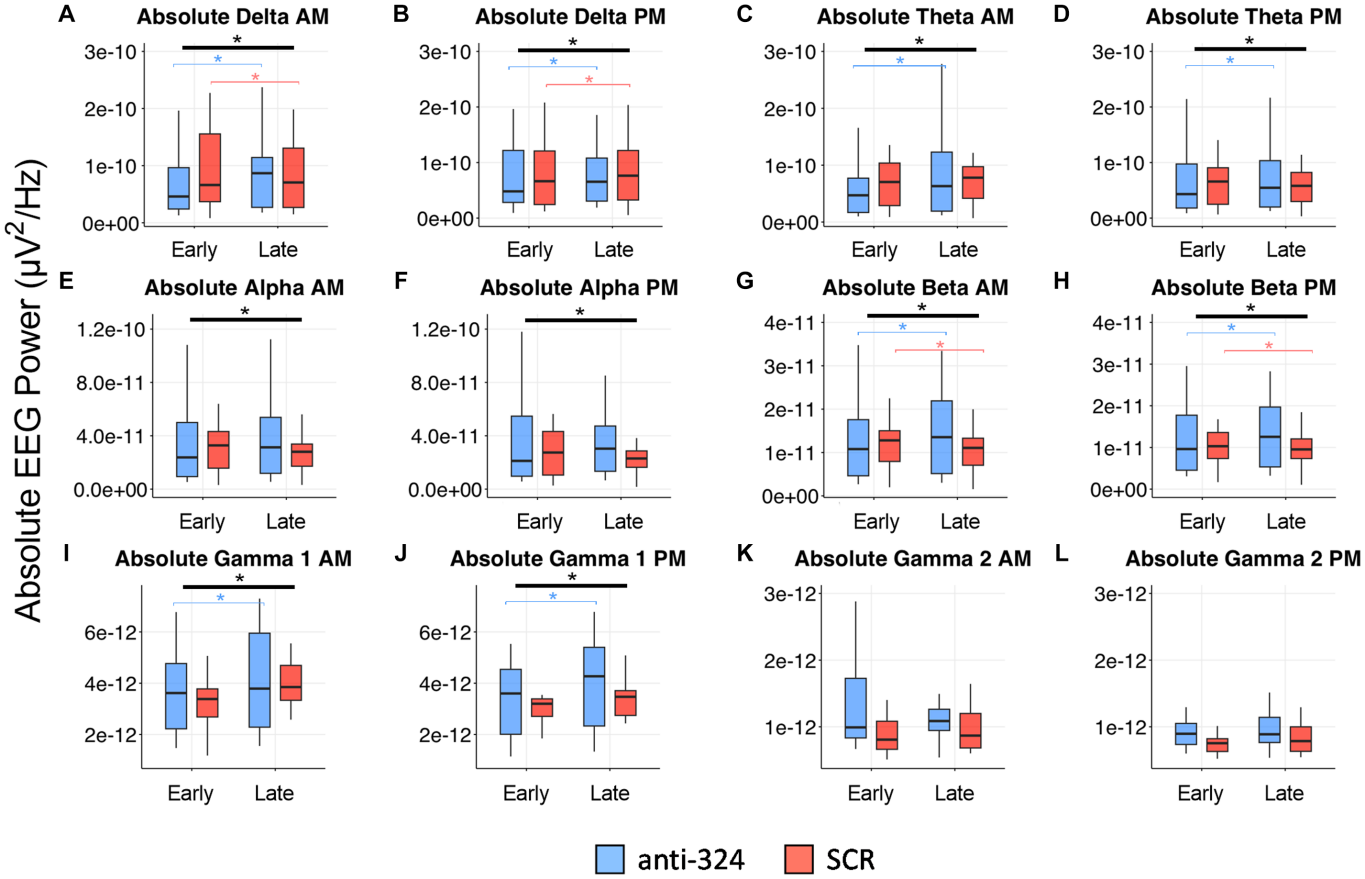
MiR-324-5p inhibition during epileptogenesis alters EEG spectral absolute power bands. Box plots show absolute power for each treatment group (anti-324 vs. SCR) during early (days 7–9) and late (days 25–27) timepoints. Data is plotted separately for a.m. and p.m. timepoints within each power band. Power band graphs are shown in order of ascending frequency: **(A)** absolute delta a.m., **(B)** absolute delta p.m., **(C)** absolute theta a.m., **(D)** absolute theta p.m., **(E)** absolute alpha a.m., **(F)** absolute alpha p.m., **(G)** absolute beta a.m., **(H)** absolute beta p.m., **(I)** absolute gamma 1 a.m., **(J)** absolute gamma 1 PM, **(K)** absolute gamma 2 a.m., **(L)** absolute gamma 2 p.m. See sections Results and Materials and methods for details on data analysis and graphical representation. Box plots are for power data visualization, not statistical analysis. Power data was statistically analyzed by RM linear mixed model, shown in Table 1 (absolute) and Supplementary Table 1 (relative). Corresponding box plots representing relative power data are shown in Supplementary Figure 3. All indications of significant data are based on linear mixed model statistics. Significant interactions represented by thick black bar with asterisk above all boxes. Significant pairwise comparisons (324 late-324 early or SCR late-SCR early) represented by thin, bracketed bars with asterisks above boxes for each treatment. Bracketed bars related to pairwise comparisons color-coded based on treatment: blue (324), orange (SCR).

Figures 4, 5, Supplementary Figure 3, Table 1, and Supplementary Table 1 only include mice with continuous 24/7 EEG data on days 7–9 and 25–27 [*n*(324) = 10, *n*(SCR) = 8].

**Table 1:** Treatment × Timepoint effects and pairwise comparisons in absolute power by linear mixed model.

Frequency band	Interaction, pairwise comparisons	Estimates	SEM	*p*
Absolute delta*2–4 Hz*	Treatment × Timepoint324 Late – 324 EarlySCR Late – SCR Early	0.033^*^0.000^*^0.036^*^	0.3730.157	0.0670.075
Absolute theta*4–10 Hz*	Treatment × Timepoint324 Late – 324 EarlySCR Late – SCR Early	0.017^*^0.001^*^0.821	0.158−0.012	0.0470.053
Absolute alpha*10–13 Hz*	Treatment × Timepoint324 Late – 324 EarlySCR Late – SCR Early	0.010^*^0.0600.075	0.089−0.094	0.0470.053
Absolute beta*13–30 Hz*	Treatment × Timepoint324 Late – 324 EarlySCR Late – SCR Early	0.000^*^0.000^*^0.048^*^	0.136−0.075	0.0340.038
Absolute gamma 1^†^*30–55 Hz*	Treatment × Timepoint324 Late – 324 EarlySCR Late – SCR Early	0.024^*^0.000^*^0.434	0.5590.114	0.1300.146
Absolute gamma 2*65–100 Hz*	Treatment × Timepoint324 Late – 324 EarlySCR Late – SCR Early	0.6740.7590.401	0.0150.045	0.0470.053

Spectral power analysis at early (days 7–9) and late (days 25–27) timepoints showed significant interactions between treatment and timepoint regardless of period (AM/PM) for distinct EEG frequency bands, suggesting that treatment altered disease progression. Shown are results of a RM linear mixed model fitted for each power band. Power bands were log transformed with the exception of absolute gamma 1, which was not transformed (†). Estimates represent absolute power multiplied by 10^12^. *p < 0.05. Pairwise comparisons not listed were not statistically significant. See Results and Materials and methods text details on analysis. Corresponding results for relative power are shown in Supplementary Table 1.

## Results

### Intrahippocampal kainic acid injection reliably induces *status epilepticus* and initiates epileptogenesis

Based on its anti-epileptic effects on acute and chronic seizures, we hypothesized that miR-324-5p antagonism during epileptogenesis would delay or inhibit the development of epilepsy and related symptoms. To test this hypothesis, we used the mouse intrahippocampal kainic acid (IHpKa) model of temporal lobe epilepsy. We selected this model for its reported reliable induction of *status epilepticus* (*SE*), localized brain insult, latent period greater than 2 days (to allow for miRNA antagonism following epileptogenic insult), and low mortality (42). The experimental design and timeline are shown in Figure 1A. Briefly, mice were intrahippocampally injected with kainic acid or saline on day 0. Twenty-four hours after the epileptogenic insult, mice were injected intracerebroventricularly with a miR-324-5p (anti-324) or scrambled control (SCR) antagomir. Antagomir treatment was administered 24 h after *SE* induction specifically to ensure antagonism of miR-324-5p during the latent period in this model. Mice were implanted with cortical electrodes for 24/7 EEG monitoring on either day 0 (during kainic acid administration) or day 1 (during antagomir injection). We then recorded 24/7 video-EEG for 28 days, at which point brain tissue was collected for immunohistochemical analysis.

In most mice, we confirmed initial *SE* behaviorally based on the occurrence of multiple Racine class 5 seizures within 24 h of IHpKa. In a subset of mice, we implanted cortical EEG electrodes on day 0 (rather than day 1) to electrographically confirm initial *SE*. Data show IHpKa reliably induced electroencephalographic *SE* within 24 h of administration, while control animals injected with saline did not show epileptic activity (example EEG tracings shown in Figure 1B). The average latency to EEG-confirmed *SE* was 25.4 ± 2.0 (SEM) minutes and the average duration of *SE* was 11.2 ± 1.9 (SEM) hours. At the completion of the study, we analyzed latency and duration of initial *SE* on day 0 in mice that were subsequently placed into the two treatment groups (anti-324 vs. SCR) on day 1 to ensure mice had not been inadvertently biased toward either treatment group based on initial *SE* severity. There was no significant difference in the latency to or duration of initial *SE* between mice subsequently assigned to the anti-324 or SCR treatment group, supporting lack of experimental bias in group assignment [Figures 1C,D, respectively; unpaired *t*-tests, C: *t* = 1.62, *p* = 0.15, *n*(anti-324) = 5, *n*(SCR) = 4; D: *t* = 0.81, *p* = 0.44, *n*(anti-324) = 5, *n*(SCR) = 4].

### Antagomir treatment 24 h after intrahippocampal kainic acid does not affect long-term survival rate

Over 90% of mice survived the experimental protocol regardless of treatment, consistent with mortality reports in this model (42) (Figure 1E). Antagomir treatment had no significant effect on overall survival [Mantel-Cox test: X^2^ = 0.02, *p* = 0.89, *n*(anti-324) = 14, *n*(SCR) = 12].

### Antagomir treatment does not prevent granule cell dispersion or pyramidal cell loss

Intrahippocampal kainic acid administration in mice causes dentate granule (DG) cell dispersion and CA1 cell death in the hippocampus ipsilateral to the injection site (43–47). To further validate the model and determine whether antagomir treatment affected IHpKa-associated morphological changes, we assessed hippocampal morphology 28 days after kainic acid injection using NeuN immunohistochemical staining (representative images in Figure 2A). Only mice that survived to day 28 were used in immunohistochemical analyses. We visually screened up to four serial sections (average = 3 sections, range = 1–4 sections) per mouse between Bregma levels −1.06 mm and − 3.28 mm to assess morphological changes along the dorsoventral axis. A more detailed analysis of the incidence of morphological changes by treatment across the dorsoventral brain axis can be found in Supplementary Figure 2. Overall, the proportion of mice that exhibited any hippocampal damage (DG dispersion and/or CA1 cell death; Figure 2B), DG dispersion (Figure 2C), or CA1 cell death (Figure 2D) did not differ between antagomir treatment groups [Fisher’s exact tests, *p* > 0.05, *n*(anti-324) = 9, *n*(SCR) = 9].

To grossly quantify DG dispersion, we determined the total dentate gyrus area and the area of DG cell body layers ipsi- and contralateral to the kainic acid injection site (representative tracings to determine areas in Figure 2E). Consistent with the IHpKa model, the kainic acid-injected (ipsilateral) dentate gyrus was larger than the non-injected (contralateral) dentate gyrus [Figure 2G, two-way ANOVA: *p*(interaction) = 0.26, *p*(side) = 0.02, *p*(treatment) = 0.27, *n*(anti-324) = 9, *n*(SCR) = 9]. Similarly, independent of treatment, the area of the ipsilateral DG cell layer was larger than that of the contralateral [Figure 2H, two-way ANOVA: *p*(interaction) = 0.19, *p*(side) = 0.004, *p*(treatment) = 0.20, *n*(anti-324) = 9, *n*(SCR) = 9]. To approximate dispersion of the DG cell layer, we expressed the area of that layer as a percentage of the total area of the dentate gyrus. Analysis of this dispersion value showed comparable results: independent of treatment, the extent of DG cell layer dispersion was greater in the dentate gyrus ipsilateral to kainic acid injection than in the contralateral dentate gyrus [Figure 2I, two-way ANOVA: *p*(interaction) = 0.37, *p*(side) = 0.0007, *p*(treatment) = 0.35, *n*(anti-324) = 9, *n*(SCR) = 9].

To approximate cell death in the CA1, we measured the total length of the CA1 pyramidal cell layer and the length of the pyramidal cell layer with positive NeuN-staining (representative tracings to measure lengths in Figure 2F). The total length of the CA1 pyramidal cell layer did not differ by treatment nor by laterality [two-way ANOVA: *p*(interaction) = 0.33, *p*(side) = 0.39, *p*(treatment) = 0.55, *n*(anti-324) = 9, *n*(SCR) = 9, *data not shown*]. Similar to the DG dispersion results, CA1 cell death was noticeably greater in the hippocampus ipsilateral to kainic acid injection than in the contralateral, independent of treatment [Figure 2J, two-way ANOVA: *p*(interaction) = 0.71, *p*(side) < 0.0001, *p*(treatment) = 0.98, *n*(anti-324) = 9, *n*(SCR) = 9].

Overall, our results show that IHpKa results in DG dispersion and CA1 cell death ipsilateral to IHpKa independent of antagomir treatment.

### MiR-324-5p inhibition during epileptogenesis does not alter seizure incidence

A representative electroencephalographic SRS collected via cortical electrodes is shown in Figure 3A. Antagomir treatment had no effect on the duration of the latent period [time to first SRS; Figure 3B, Mantel-Cox test: X^2^ = 0.10, *p* = 0.76, *n*(anti-324) = 6, *n*(SCR) = 8]. Seizure latency for both groups aligned with previous reports regarding the latent period in this model (2-14d) (42); the median latent period for mice treated with anti-324 and SCR were 8 and 11.5 days, respectively.

Treatment also did not affect SRS frequency over the 28-day period following IHpKa [Figure 3C, two-way ANOVA: *p*(interaction) = 0.96, *p*(day) = 0.15, *p*(treatment) = 0.57, *n*(anti-324) = 9–13, *n*(SCR) = 9–14]. Notably, mice treated with IHpKa typically have 1–2 cortical SRSs per week (42). Because our data aligned closely with that measure, many mice in both treatment groups had zero SRSs on several individual days. To account for the low overall daily seizure frequency, we analyzed total seizures by week, focusing on an early time point (week 1, days 1–7) and a later time point (week 4, days 22–28) to account for the expected progressive nature of this model. Total SRSs in week 1 and week 4 did not statistically differ by week nor antagomir treatment group [Figure 3D, two-way ANOVA: *p*(interaction) = 0.50, *p*(week) = 0.08, *p*(treatment) = 0.14, *n*(anti-324, Week 1) = 10, *n*(anti-324, Week 4) = 10, *n*(SCR, Week 1) = 10, *n*(SCR, Week 4) = 8]; however, the proportion of mice in each treatment group that experienced SRSs in week 4 was greater than that of week 1, as expected in this progressive model. Antagomir treatment also had no significant effect on seizure burden [total time spent seizing; Figure 3E, two-way ANOVA: *p*(interaction) = 0.77, *p*(week) = 0.08, *p*(treatment) = 0.17, *n*(anti-324, Week 1) = 10, *n*(anti-324, Week 4) = 10, *n*(SCR, Week 1) = 10, *n*(SCR, Week 4) = 8]. Antagomir treatment did not significantly affect average seizure duration [Figure 3F, anti-324: 40.80 ± 3.69 s, SCR: 33.00 ± 3.85 s, unpaired *t*-test: *t* = 1.46, *p* = 0.18, *n*(anti-324) = 5, *n*(SCR) = 5]. Regardless of antagomir treatment, the majority of SRSs were behavioral seizures of class 4/5 or 5/5 on the Racine Scale (37) [anti-324: 4.32 ± 0.10, SCR: 4.22 ± 0.92, unpaired *t*-test: *p* = 0.49, *n*(anti-324) = 119 seizures, *n*(SCR) = 175 seizures, *data not shown*]. Overall, our data show that antagonism of miR-324-5p does not significantly affect SRS latency, frequency, or duration within 28 days of IHpKa.

### MiR-324-5p inhibition during epileptogenesis does not alter frequency of interictal spikes or spike trains

To further investigate the effect of miR-324-5p antagonism on the formation of an epileptic network, we analyzed interictal spikes and spike trains using 24/7 cortical EEG recordings. A representative spike collected via cortical electrodes is shown in Figure 4A, and a representative spike train is shown in Figure 4C. There was no difference in spike frequency based on antagomir treatment or timepoint [Figure 4B, two-way RM ANOVA: *p*(interaction) = 0.59, *p*(timepoint) = 0.44, *p*(treatment) = 0.61, *n*(anti-324) = 10, *n*(SCR) = 8]. There was also no difference in spike train frequency based on antagomir treatment or timepoint [Figure 4D, two-way RM ANOVA: *p*(interaction) = 0.17, *p*(timepoint) = 0.45, *p*(treatment) = 0.60, *n*(anti-324) = 10, *n*(SCR) = 8]. To account for variability in spike frequency, we calculated the within-subject percentage change in spikes between early and late timepoints. Antagomir treatment had no effect on percentage change in spike frequency over time [Figure 4E, Mann–Whitney test: *p* = 0.74, *n*(anti-324) = 10, *n*(SCR) = 8]. Overall, our data suggest that antagomir treatment does not affect development of interictal spikes or spike trains within the first 4 weeks after IHpKa.

### MiR-324-5p inhibition during epileptogenesis alters EEG spectral power bands

In addition to interictal spike analysis, we investigated the effect of miR-324-5p antagonism during epileptogenesis on EEG spectral power. Absolute EEG power data by treatment and timepoint are visually represented in Figure 5 (relative power in Supplementary Figure 3). We assessed the interaction of treatment (anti-324 vs. SCR) and timepoint (early vs. late) for each frequency band using repeated-measures linear mixed models [Table 1, *n*(324 Late–324 Early) = 30, *n*(SCR Late–SCR Early) = 24]. Note Figure 5 is for visual representation of power data only; statistical analyses were performed using repeated-measures linear mixed models. All significant findings based on linear mixed models are shown in Table 1. Our results show significant interactions between treatment and timepoint for most, but not all frequency bands. Notably, significant interactions and pairwise comparisons were significant regardless of time of day. Overall, our results indicate that mice treated with anti-324 had more pronounced increases from early to late time points in absolute delta, theta, beta, and gamma 1 power than mice treated with SCR. Absolute delta power increased over time in both treatment groups, with a greater increase in anti-324-treated mice. Absolute theta and gamma 1 power increased in anti-324-treated mice and did not change in SCR-treated mice. While absolute beta power increased in mice treated with anti-324, it decreased in mice treated with SCR. No significant interactions were detected for absolute gamma 2. Pairwise comparisons not reported showed no significance. There were no significant interactions for relative frequency bands (Supplementary Figure 3). Overall, our data show that inhibition of miR-324-5p during epileptogenesis altered the temporal development of frequency band-specific EEG power.

## Discussion

The main finding of this study is that inhibition of miR-324-5p during the latent period after an epileptogenic insult does not prevent epilepsy development in mice. This is in contrast to our previous studies, which showed that miR-324-5p inhibition in mice reduces seizure susceptibility when administered shortly before brain insult and reduces seizure frequency when administered during chronic epilepsy (21, 23). Despite the anti-convulsant effects of miR-324-5p at other disease stages, this study demonstrates that miR-324-5p inhibition during epileptogenesis induced by intrahippocampal kainic acid does not significantly affect several important hallmarks of epilepsy: hippocampal damage, SRS latency, frequency, or duration, or interictal spike frequency. MiR-324-5p inhibition during epileptogenesis does, however, alter EEG power band changes over time. Ultimately, this suggests that miR-324-5p may act as a proconvulsive rather than a pro-epileptogenic regulator. In other words, miR-324-5p inhibition may alleviate epilepsy symptoms when administered in close temporal proximity to seizure – shortly before an epileptogenic insult (*SE*) or in chronic epilepsy when SRSs occur – but it is not effective at preventing the development of epilepsy when administered during epileptogenesis. Supporting its role as a proconvulsant miRNA, miR-324-5p targets and downregulates the A-type potassium channel Kv4.2 (21, 23). Reduction or deletion of Kv4.2 increases susceptibility to pharmacologically-induced seizures but does not lead to the development of spontaneous seizures (48, 49), suggesting that the channel is not directly involved in epileptogenesis. While miRNAs can target a large network of mRNAs (11), our results combined with these previous findings suggest that the impact of miR-324-5p on epilepsy depends largely on its acute regulation of network excitability.

Despite the lack of effects on seizure latency and frequency, we detected changes in the temporal development of frequency band-specific EEG power. We found that absolute delta power increased over time to a greater extent in mice treated with anti-324 than in those treated with SCR. Delta power has been shown to be increased in rodent models of epilepsy and human epilepsy patients (50–52), suggesting that antagonism of miR-324-5p may actually have resulted in a more epileptic network than control treatment. Similarly, absolute theta power increased in mice treated with anti-324. Theta power has been shown to be higher in human epilepsy patients (51), which again could be interpreted as a pro-epileptic effect of miR-324-5p inhibition. Mice treated with anti-324 had increased absolute beta power over time, while mice treated with SCR had decreased beta power over time. A similar trend occurred for absolute alpha power. Mice treated with anti-324 also had significant increases in absolute gamma 1 power over time. There is conflicting data on the alterations in alpha, beta, and gamma 1 power bands over time in epilepsy models; indeed, there are even conflicting relationships between symptom severity and EEG power in individual human epilepsy patients (51, 53–55). Interpretation of the observed differences in EEG power with regard to their relevance for epileptogenesis is thus challenging due to limited knowledge about the roles and contributions of specific frequency power bands in epilepsy. It is important to note that we observed significant differences in absolute but not relative EEG power. Absolute power could be affected by overall signal strength; however, given the relatively large sample size and the fact that anti-324 and SCR mice were run and analyzed in parallel, it is unlikely that observed effects are due to biases in overall signal strength. Interpretation of spectral power in this study is limited by the inability of our video-EEG system to differentiate sleep from wake in mice. EEG power is known to vary based on sleep–wake cycles (56). While we analyzed data in non-moving mice, our experimental set-up did not allow for distinguishing specific sleep and awake states, and it is thus unclear whether mice were awake or asleep in each EEG analysis segment. Importantly, we analyzed EEG recordings during both a.m. and p.m. time periods and found no significant differences in spectral power between those time periods. Moreover, to prevent any inadvertent bias toward a specific sleep or awake state, we analyzed three periods of 5 min over three consecutive days during which mice were not moving, allowing us to capture multiple sleep/awake stages in the mice. Given a number of frequency bands changed with no clear pattern in their relation to epileptogenesis, it is likely most appropriate to conclude that normal brain networks become altered following kainic acid injection and that miR-324-5p affects these alterations over time. Whether this effect is relevant for epileptogenesis is unclear. Longer-term studies are needed to further address this question.

Histological analyses showed that IHpKa induced characteristic hippocampal damage (dentate granule cell layer dispersion and CA1 cell death) at the injection site independent of antagomir treatment (42) (Figure 2), and that miR-324-5p inhibition had no effect on morphological outcomes (Supplementary Figure 2). This suggests that miR-324-5p does not significantly contribute to early morphological changes during epileptogenesis. The prominent damage to the hippocampus ipsilateral to kainic acid injection and the relative absence of damage to the contralateral hippocampus suggest that hippocampal damage is likely primarily due to the excitotoxicity of kainic acid rather than resultant generalized seizure activity. Of note, total SRSs over 28 days following IHpKa did not correlate with hippocampal damage (*data not shown*). Future studies are needed to elucidate the mechanisms dictating disease severity, which may be more subtle morphological or molecular alterations.

Our results indicate that miR-324-5p inhibition had no significant effect on epileptogenesis, but we acknowledge that the study had several limitations. First, to initiate epileptogenesis in mice, we used the intrahippocampal kainic acid model of temporal lobe epilepsy. The model consistently initiated epileptogenesis via induction of *SE*, and our measures of *SE* duration, latent period duration, frequency of spontaneous recurrent seizures, and overall mortality closely aligned with others in the literature (42) (Figure 1). Importantly, the latent period of this model allowed for inhibition of miR-324-5p during epileptogenesis before the development of chronic epilepsy (Figure 3B). Like many epilepsy models, the IHpKa model shows high variability between mice. While this is useful in modeling the variability seen in human epilepsy patients, it also complicates data interpretation, leading to high within-group variability. The goal of this study was to assess the effects of antagomir treatment during epileptogenesis (first 4 weeks after *SE*); however, due to the progressive nature of this model (46, 47, 57), it is possible that longer-term analysis would have revealed group differences that did not appear in the first 4 weeks following IHpKa. In addition, we used cortical rather than hippocampal depth electrodes to collect EEG data. Cortical electrodes did not allow for the detection of focal hippocampal seizures, which most likely precede or accompany generalized seizures, or other characteristic EEG changes associated with this model, such as hippocampal paroxysmal discharges and high frequency oscillations (33, 36, 57). It is possible that use of subdural electrodes may have uncovered subtle antagomir treatment effects. Nevertheless, the reliable initiation of epileptogenesis combined with the low mortality rate compared with other epilepsy models (58) made the IHpKa model appropriate for assessing antagomir treatment during epileptogenesis, and useful in long-term analysis of epilepsy symptoms. Future studies could explore if miR-324-5p regulates epileptogenesis after other forms of brain insults, such as traumatic brain injury.

Second, we administered only a single antagomir dose at one timepoint and did not have a direct read-out of antagomir efficacy. We chose the dose based on our previous studies showing an anti-convulsant effect (21, 23). To our knowledge, the effect of different dosages has not been investigated with this antagomir. Other studies have shown that antagomir dosage greatly affects target mRNA and protein levels (59, 60); thus, it is possible that an increased dosage or continuous administration (i.e., via osmotic pump) would have produced a treatment effect. Antagomirs have been shown to function for several weeks in the periphery and brain, albeit with reduced activity (61, 62). Although non-significant for SRSs, mice treated with anti-324 appeared to have larger epileptogenic changes from early to late timepoints than SCR-treated mice (Figures 3, 5; Table 1). This could be due to reduced levels of active anti-324 over time; thus, it is possible that subsequent or continuous doses of antagomir would have amplified between-group differences. While this study did not include a direct read-out of antagomir efficacy, it did provide indirect proof through the observed differences in spectral power between treatment groups. Moreover, previous studies in our lab and others have confirmed functional activity of the miR-324-5p antagomir at 24 h post-injection via changes in target gene expression and seizure susceptibility (21), at 7–14 days post-injection via changes in A-type currents and seizure susceptibility (23), and at 2 weeks post-injection via changes in dendritic spine morphology (63).

Third, given the complexity of miRNA involvement in epilepsy, inhibition of only one miRNA may not be sufficient to inhibit epileptogenesis. In the future, the manipulation of miR-324-5p and multiple other epilepsy-related miRNAs simultaneously may have a larger effect on epileptogenesis. Indeed, others have successfully ameliorated epilepsy symptoms using combined antagomirs to inhibit several miRNAs simultaneously (64).

Fourth, only male mice were assessed in this study. Reproductive hormones are known to affect epilepsy in a cyclical manner (65) and miR-324-5p targeting of Kv4.2 correlates with female sex hormone levels in peripheral blood (24); we thus used only male mice to limit variability. Nonetheless, it will be important to include both male and female mice in future work, as epilepsy affects both male and female patients.

Despite these limitations, there are several important conclusions that can be drawn from this study, which will inform future research into the therapeutic potential of miRNA-based treatments in epilepsy. Most importantly, this study underscores the significance of timepoint of treatment in epilepsy. While inhibition of miR-324-5p decreased seizure severity when administered before brain insult or in chronic epilepsy (21, 23), the same treatment paradigm did not exert strong effects when administered during epileptogenesis. Ongoing studies in the lab aim to clarify the functional engagement of miR-324-5p across several timepoints of epilepsy development as well as the mRNA network targeted by miR-324-5p during this period. A clearer understanding of its timepoint-specific engagement and target mRNA network may clarify the effect of miR-324-5p inhibition at each stage of epilepsy development. It is critical to assess treatment efficacy during epileptogenesis because treatment during this timepoint may prevent or delay additional symptoms. Understanding of successful interventions during epileptogenesis could allow patients to be identified based on history of brain insult and treated before the development of chronic epilepsy. Importantly, epileptogenesis does not end with onset of spontaneous recurrent seizures; every seizure initiates additional epileptogenic brain changes (2). Thus, treatments that inhibit epileptogenesis could benefit patients in both the latent and chronic stages of the disease.

## Data availability statement

The raw data supporting the conclusions of this article will be made available by the authors, without undue reservation.

## Ethics statement

The animal study was approved by Institutional Animal Care and Use Committee, Cincinnati Children’s Hospital Medical Center. The study was conducted in accordance with the local legislation and institutional requirements.

## Author contributions

AM: Data curation, Formal Analysis, Investigation, Visualization, Writing – original draft. GW: Formal Analysis, Writing – review & editing. AC: Formal Analysis, Investigation, Writing – review & editing. CD: Formal Analysis, Writing – review & editing. EPa: Investigation, Writing – review & editing. VR: Investigation, Writing – review & editing. PH: Writing – review & editing, Formal Analysis. EPe: Formal Analysis, Methodology, Writing – review & editing. DT: Methodology, Writing – review & editing. SD: Writing – review & editing, Conceptualization. CG: Conceptualization, Formal Analysis, Funding acquisition, Methodology, Writing – review & editing.
