# Supplementary material for: MiR-324-5p inhibition after intrahippocampal kainic acid-induced status epilepticus does not prevent epileptogenesis in mice
Source: Front Neurol. 2023 Nov 16;14:1280606. doi: 10.3389/fneur.2023.1280606 (PMC10687438; doi:10.3389/fneur.2023.1280606)
Supplement: Supplementary file 1 [file Data_Sheet_1.pdf]

Supplementary Material

MiR-324-5p inhibition after intrahippocampal kainic acid-induced *status epilepticus* does not prevent epileptogenesis in mice

McGann, AM; Westerkamp, GC; Chalasani, A; Danzer, CSK; Parkins, EV; Rajathi, V; Horn, PS; Pedapati, EV; Tiwari, D; Danzer, SC; Gross, C

*** Correspondence:** Christina Gross: Christina.Gross@cchmc.org

Supplementary Figures and Tables

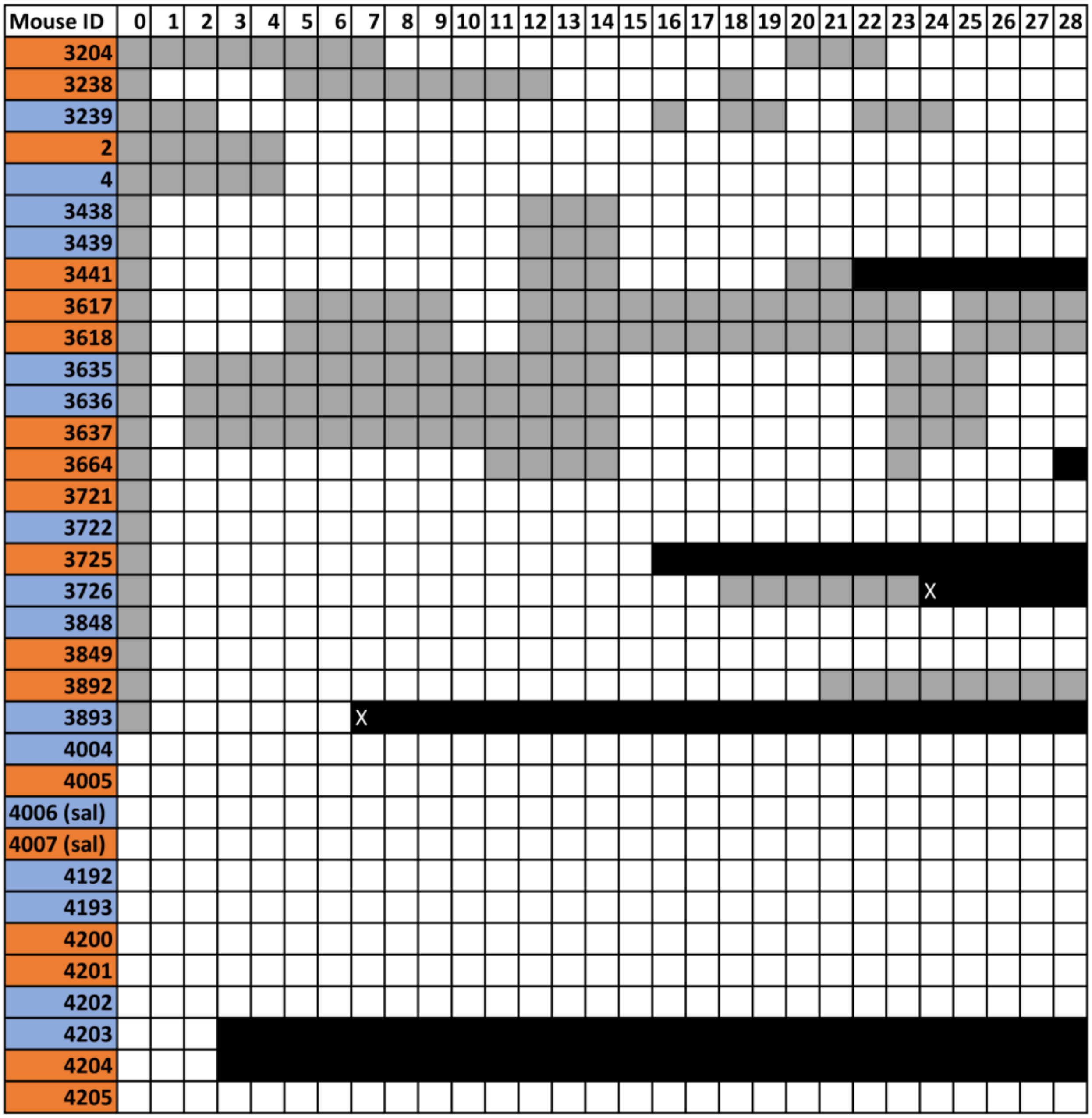

Supplementary Figure 1: Availability of EEG data by mouse. Table outlines availability of EEG data by mouse (leftmost column) and day (0-28). "Sal" indicates treatment with intrahippocampal saline rather than kainic acid (n = 2). Background of "Mouse ID" indicates antagomir treatment: anti-324 = blue, SCR = orange. White boxes indicate available EEG, gray boxes indicate missing EEG data from living mouse, black boxes indicate deceased mouse. The "X" marker indicates death related to epilepsy model.

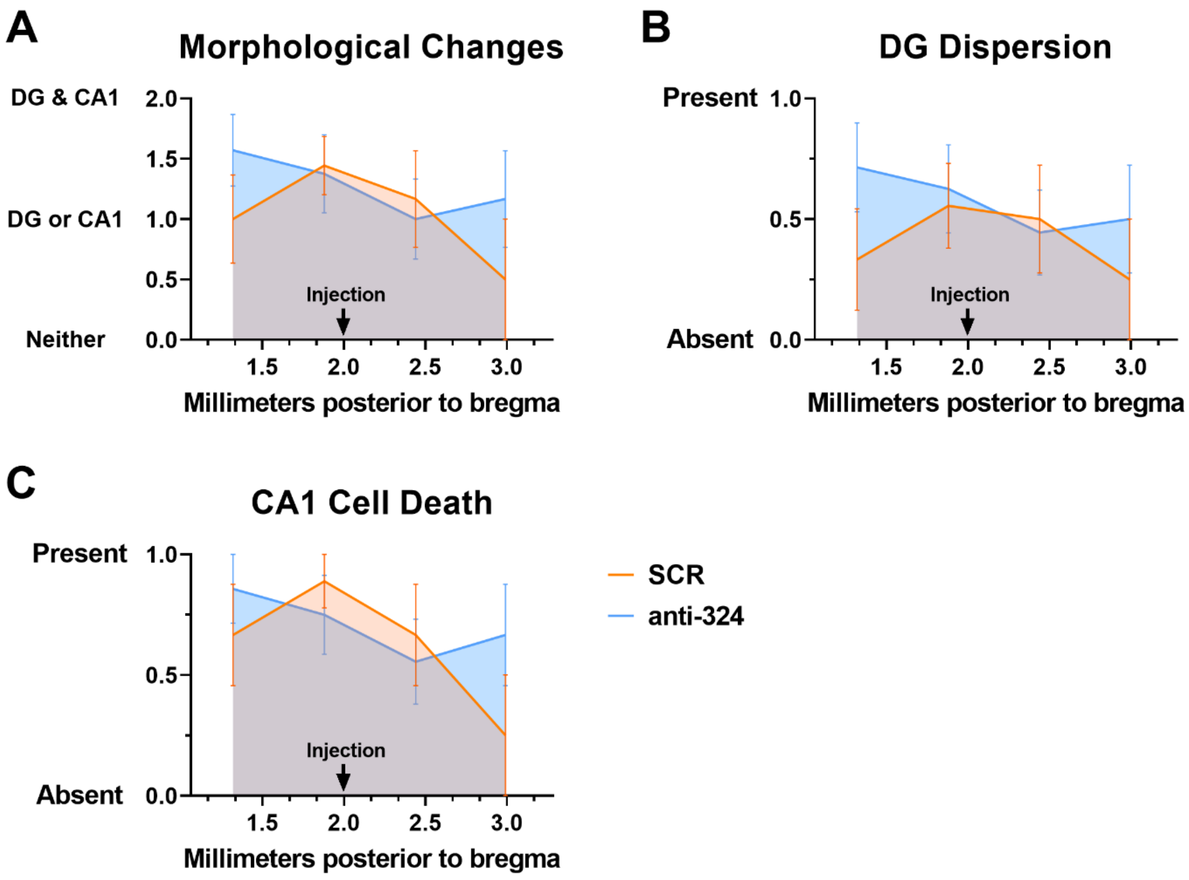

Supplementary Figure 2: Antagomir treatment does not affect incidence of granule cell dispersion and/or pyramidal cell loss along the dorsoventral brain axis. Up to four serial sections (average = 3 sections, range = 1-4 sections) per mouse between Bregma levels -1.06mm and -3.28mm were visually assessed for the presence of morphological changes. Sections were binned into one of four Bregma levels: -1.06mm to -1.58mm (n(anti-324) = 7, n(SCR) = 6), -1.58mm to -2.18mm (n(anti-324) = 8, n(SCR) = 9), -2.18mm to -2.70mm (n(anti-324) = 9, n(SCR) = 6), and -2.70mm to -3.28mm (n(anti-324) = 6, n(SCR) = 4). **(A)** To assess overall morphological changes along the dorsoventral brain axis, each section was scored “0” if it did not exhibit DG dispersion or CA1 cell death, “1” if it exhibited either DG dispersion or CA1 cell death, and "2" if it exhibited both DG dispersion and CA1 cell death. Antagomir treatment did not affect overall incidence of morphological changes (Two-way mixed ANOVA: p(interaction) = 0.31, p(level) = 0.10, p(treatment) = 0.42, n(anti-324) = 6-9, n(SCR) = 4-9). **(B, C)** To assess incidence of DG dispersion and CA1 cell death along the dorsoventral brain axis, sections were scored “0” if they did not exhibit DG dispersion (B) or CA1 cell death (C) and “1” if they did exhibit DG dispersion or CA1 cell death. Antagomir treatment did not affect incidence of DG dispersion (B, two-way mixed ANOVA: p(interaction) = 0.64, p(level) = 0.32, p(treatment) = 0.52, n(anti-324) = 6-9, n(SCR) = 4-9) or CA1 cell death (C, two-way mixed ANOVA: p(interaction) = 0.18, p(level) = 0.06, p(treatment) = 0.66, n(anti-324) = 6-9, n(SCR) = 4-9).

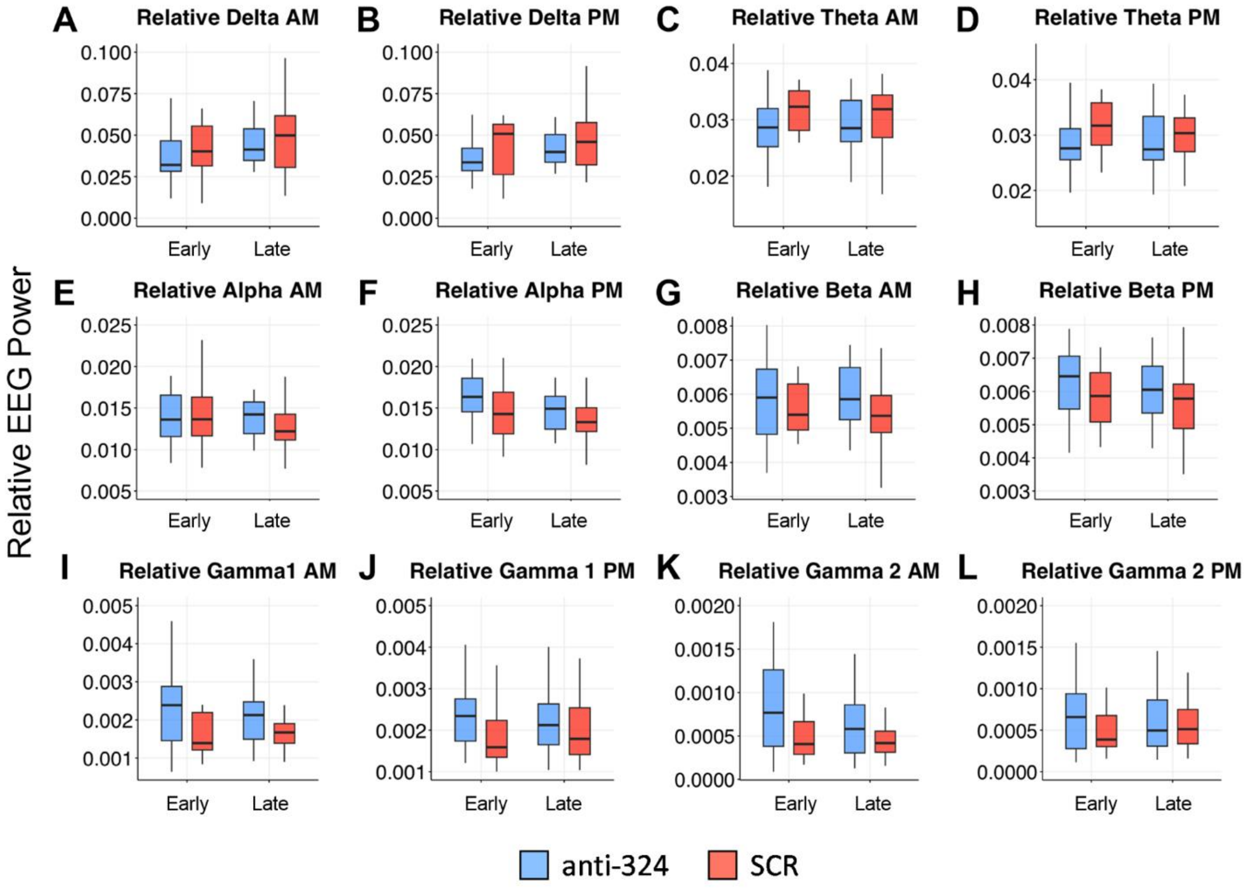

Supplementary Figure 3: MiR-324-5p inhibition during epileptogenesis does not alter EEG spectral relative power bands. Box plots show relative power for each treatment group (anti-324 vs. SCR) during early (days 7-9) and late (days 25-27) timepoints. Relative power was defined as the band-specific absolute power relative to total absolute power. Data is plotted separately for AM and PM timepoints within each power band. Power bands graphs are listed in order of ascending frequency: **(A)** relative delta AM, **(B)** relative delta PM, **(C)** relative theta AM, **(D)** relative theta PM, **(E)** relative alpha AM, **(F)** relative alpha PM, **(G)** relative beta AM, **(H)** relative beta PM, **(I)** relative gamma 1 AM, **(J)** relative gamma 1 PM, **(K)** relative gamma 2 AM, **(L)** relative gamma 2 PM. See results and methods for details on data analysis and graphical representation. Box plots are for power data visualization, not statistical analysis. Power data was statistically analyzed by RM linear mixed model, shown in Table 1 (absolute) and Supplementary Table 1 (relative).

Frequency Band	Interaction, Pairwise Comparisons	Estimates	SEM	*p*
Relative delta†	Treatment x Timepoint			0.829
2-4Hz	324 Late - 324 Early	72.428	15.949	0.000*
	SCR Late - SCR Early	77.600	17.832	0.000*
Relative theta†	Treatment x Timepoint			0.653
4-10Hz	324 Late - 324 Early	0.718	4.225	0.865
	SCR Late - SCR Early	-2.138	4.724	0.651
Relative alpha	Treatment x Timepoint			0.780
10-13Hz	324 Late - 324 Early	-0.063	0.029	0.031*
	SCR Late - SCR Early	-0.075	0.032	0.021*
Relative beta†	Treatment x Timepoint			0.455
13-30Hz	324 Late - 324 Early	-1.523	1.006	0.132
	SCR Late - SCR Early	-2.653	1.125	0.019*
Relative gamma 1	Treatment x Timepoint			0.701
30-55Hz	324 Late - 324 Early	-0.022	0.038	0.556
	SCR Late - SCR Early	0.000	0.042	0.991
Relative gamma 2	Treatment x Timepoint			0.083
65-100Hz	324 Late - 324 Early	-0.174	0.055	0.002*
	SCR Late - SCR Early	-0.031	0.061	0.608

Supplementary Table 1: Treatment x timepoint effects and pairwise comparisons in relative power by linear mixed model. Spectral power analysis at early (days 7-9) and late (days 25-27) timepoints showed no significant interactions between treatment and timepoint for relative EEG frequency bands. Shown are results of a RM linear mixed model fitted for each power band. Relative alpha, gamma 1, and gamma 2 bands were log transformed; relative delta, theta, and beta bands were not transformed (†). Estimates represent relative power multiplied by 10^4.
